# Draft Genome Sequence of *Anaerosphaera* sp. Strain GS7-6-2, a Coccal Bacterium Isolated from a Biogas-Related Environment

**DOI:** 10.1128/MRA.00205-19

**Published:** 2019-04-25

**Authors:** Regina Rettenmaier, Klaus Neuhaus, Wolfgang Liebl, Vladimir V. Zverlov

**Affiliations:** a Department of Microbiology, Wissenschaftszentrum Weihenstephan, Technical University of Munich, Freising, Germany; b Core Facility Microbiome/NGS, ZIEL–Institute for Food & Health, Technical University of Munich, Freising, Germany; c Institute of Molecular Genetics, RAS, Moscow, Russia; University of Maryland School of Medicine

## Abstract

Strain GS7-6-2 was isolated from a mesophilically operated biogas fermenter. The 16S rRNA gene sequence (93.27% identity to Anaerosphaera aminiphila WN036^T^) indicated that GS7-6-2 represents a putative novel species within the genus Anaerosphaera (family *Peptoniphilaceae*). Here, we report the draft genome sequence of GS7-6-2 as established by Illumina paired-end sequencing.

## Abstract (precis)

Strain GS7-6-2 was isolated from a mesophilically operated biogas fermenter. The 16S rRNA gene sequence (93.27% identity to Anaerosphaera aminiphila WN036^T^) indicated that GS7-6-2 represents a putative novel species within the genus Anaerosphaera (family *Peptoniphilaceae*).

## ANNOUNCEMENT

Organisms within the family *Peptoniphilaceae* (phylum *Firmicutes*, class *Tissierellia*, order *Tissierellales*) were recently defined as a group of organisms belonging to the genera Anaerococcus, Anaerosphaera, Helcococcus, Finegoldia, Gallicola, Murdochiella, and Parvimonas (1). The species of this family are in general described as asaccharolytic, but they metabolize peptone and amino acids to produce mainly butyrate, acetate, and lactate (2). Most of these organisms have been isolated from the human intestine or clinical material (1). Remarkably, Anaerosphaera aminiphila WN036^T^ (3) was until now the only species within this family isolated from a methanogenic cattle manure reactor. In this study, we report the draft genome sequence of GS7-6-2 (=DSM107952; NCBI accession no. SAMN10397725), isolated from a mesophilically operated biogas fermenter fed with maize silage, as established by Illumina paired-end sequencing.

For sequencing of the Anaerosphaera sp. strain GS7-6-2 genome, 1 µg chromosomal DNA of a clonal pure culture, cultivated anaerobically in GS2 medium (4) at 30°C, was used to prepare a DNA library using the TruSeq DNA PCR-free sample preparation kit (Illumina, Inc., San Diego, CA, USA). A protocol optimized for DNA shearing and fragment size selection was applied (5). The library was sequenced using the Illumina HiSeq system with about 3.2 million 2 × 150-bp reads in paired-end mode, according to the manufacturer’s instructions. The sequence data were assembled using Unicycler version 4.6, resulting in 42 contigs with an average 412-fold coverage. Default parameters were used for all software unless otherwise specified. The combined length of the 42 contigs is 2,233,588 bp, with a G+C content of 32.8 mol% and an *N*_50_ value of 135,633 bp. The NCBI Prokaryotic Genome Annotation Pipeline (6) was used for prediction and annotation of open reading frames (ORFs). Using this method, 2,116 coding sequences and 25 tRNAs were predicted. The 16S rRNA gene sequence (93.27% identity to Anaerosphaera aminiphila WN036^T^) indicated that GS7-6-2 represents a putative novel species within the genus Anaerosphaera (family *Peptoniphilaceae*) (7).

The genome sequence of Anaerosphaera sp. GS7-6-2 provided here will broaden the knowledge of the genus Anaerosphaera and the putative importance of its members in biogas reactor-related environments.

### Data availability.

This whole-genome shotgun project has been deposited at DDBJ/ENA/GenBank under the accession no. RLIH00000000. The version described in this paper is version RLIH01000000. Raw sequencing reads are available in the Sequence Read Archive under accession no. SRR8641363. The whole project is summarized in the NCBI under accession no. PRJNA504539.
